# Microclimatic temperatures increase the potential for vector-borne disease transmission in the Scandinavian climate

**DOI:** 10.1038/s41598-017-08514-9

**Published:** 2017-08-15

**Authors:** Najmul Haider, Carsten Kirkeby, Birgit Kristensen, Lene Jung Kjær, Jens Havskov Sørensen, Rene Bødker

**Affiliations:** 1 National Veterinary Institute, Technical University of Denmark, Copenhagen, Denmark; 2 Research and Development Department, Danish Meteorological Institute, Copenhagen, Denmark

## Abstract

We quantified the difference between the meteorological temperature recorded by the Danish Meteorological Institute (DMI) weather stations and the actual microclimatic temperatures at two or three different heights at six potential insect habitats. We then compared the impact of the hourly temperature on the extrinsic incubation period (EIP) of six pathogens. Finally, we developed a regression model, enabling us to predict the microclimatic temperatures of different habitats based on five standard meteorological parameters readily available from any meteorological institution. Microclimatic habitats were on average 3.5–5 °C warmer than the DMI recorded temperatures during midday and 1–3 °C cooler at midnight. The estimated EIP for five of the six microclimatic habitats was shorter than the estimates based on DMI temperatures for all pathogens studied. The microclimatic temperatures also predicted a longer season for virus development compared to DMI temperatures. Based on DMI data of hourly temperature, solar radiation, wind speed, rain and humidity, we were able to predict the microclimatic temperature of different habitats with an R^2^ of 0.87–0.96. Using only meteorological temperatures for vector-borne disease transmission models may substantially underestimate both the daily potential for virus development and the duration of the potential transmission season.

## Introduction

Temperature is a key driver of vector-borne disease transmission, as replication of arboviruses and parasites within the cold-blooded vectors are dependent on the environmental temperature^1, 2^. The extrinsic incubation period (EIP; the time interval between ingestion of the infected blood meals and the ability of the vectors to transmit the virus) and the blood meal digestion period (the time required to complete the cycle from blood feeding to egg-laying and blood feeding again) are greatly influenced by the actual temperature surrounding the vectors^3, 4^. Vector-borne disease transmission models for estimating the EIP and blood meal digestion period often use temperatures recorded by a meteorological institute^4–7^ rather than the actual microclimatic temperatures to which the vectors are exposed. The use of microclimatic temperatures in vector-borne disease transmission models has been hindered by the lack of microclimatic data.

In the present study, we define meteorological temperatures as temperatures measured at weather stations adhering to the standards given by the World Meteorological Organization (WMO), as described by the Commission for Instruments and Methods of Observation (CIMO)^8^. According to these standards, a WMO site should be representative of a large area (i.e. 100 to 1000 km^2^)^8^, and the weather station is therefore positioned far from obstacles and complex or urban terrain. To obtain a uniform temperature, recordings are made at specific heights (usually 2 m above the ground), using a white box, ensuring that the thermometer sensor is never exposed to direct sunlight. The standard meteorological temperature is therefore only one of many microclimatic temperatures within an area, and as a result, meteorological parameters observed at a WMO station cannot be fully representative of the potential geographically localised microclimate (e.g. an insect habitat). Using meteorological temperatures in vector-borne disease transmission models ignores the impact of the actual temperature in the habitat to which the insects are exposed.

The concept of microclimatic temperature was first described in the early nineteenth century by German plant ecologist Gregor Kraus who found that microclimatic temperature is different than meteorological temperature and difficult to formulate^9^. He defined microclimatic temperature as the climate of small space. Another German meteorologist Rudolf Geiger (1950) described the theoretical microclimatic temperature of different locations around the world focusing on urban habitats and habitats such as deserts, snow and forests^9^. Rudolf Geiger (1950) also described the mechanisms of heat exchange at ground surface, the warming and cooling process and relationship between ground surface temperature, and the relationship between ground surface temperature and factors affecting this temperature such as humidity, wind, and solar radiation^9^. Earlier this century, a number of articles published on microclimatic temperature^10–15^ but only a few of them were on vector-borne diseases^16, 17^. Microclimatic temperatures of Scandinavian countries are largely unknown and so is their potential role in vector-borne disease transmission.

Models used for estimation of vector-borne disease transmission are mostly based on monthly or daily average temperatures^4, 5, 18^. In reality, the vectors do not experience an “average temperature”, but are instead exposed to changing temperatures throughout the day^6^. There is a threshold temperature for most vector-borne diseases, below which a pathogen will not continue to develop in the vector. This threshold is 10.4 °C for bluetongue, 13.3 °C for Schmallenberg virus, 14 °C for Dirofilaria, 14.3 °C for West Nile virus, 15.4 °C for malaria, and 19 °C for dengue virus^4–6, 19–21^. Above these threshold temperatures, the rate of virus development and blood meal digestion varies greatly. Some pathogens develop at a higher rate immediately after the threshold temperature is exceeded, while some pathogens develop more quickly at even higher temperatures. Furthermore, some pathogens stop developing above certain temperatures (e.g. 34.4 °C for malaria; see graph S4)^16^. Using daily or monthly average temperatures therefore ignores the effects of threshold temperatures and the non-linear rate of development at increasing temperatures above the threshold values.

In cool Scandinavian climates like in Denmark, the models using monthly or daily mean temperatures could considerably underestimate the potential for vector-borne disease transmission. In Denmark, only two months of the year (July-August) have mean temperatures above the threshold for virus development^22^. However, the country experiences many hours above the threshold temperatures of viral replication in spring (May-June) and autumn (September-October), which is not apparent from the daily or monthly mean temperatures^22^.

Insects experience temperature that can be very local down to at a scale of centimeters to meters, yet most vector-borne disease transmission parameters (for example the EIP and blood meal digestion period) are analysed based on meteorological temperatures collected several kilometers away from their habitats^23^. This is because microclimatic temperature data are generally lacking, and the practical difficulties in getting microclimatic temperatures from many different habitats and heights. Studies are available on microclimatic temperatures^10–12, 17, 23^, but only some of these describe the difference between weather-station-based meteorological temperatures and microclimatic temperatures^11, 17, 23^. In these studies air temperature, humidity, solar radiation and precipitation were identified as key parameters for predicting microclimatic temperature^11, 17, 23^. Kearney *et al*. published a dataset on predicted microclimatic temperatures of three substrates (soil, rock, and sand) on a global level^10^. Such models or data are not available for terrestrial temperatures, particularly for potential insect habitats applicable to vector-borne diseases. In this study, we modelled microclimatic temperatures of six different habitats in Denmark (dry meadow, wet meadow, hedges, forest/trees, cattle grazing field and horse grazing field) based on available hourly meteorological data from the DMI, measured at weather stations adhering to WMO standards.

Denmark’s climate was previously predicted to be too cold for the spread of bluetongue virus^4^, yet the country experienced a substantial bluetongue outbreak in the years 2007 and 2008^24^. In this study, we modelled the EIP and blood meal digestion period using microclimatic temperature instead of the traditional approach of using temperatures recorded by meteorological institutes, and used hourly temperature data instead of monthly or daily mean temperatures. Our objectives were to: 1) quantify the difference between meteorological temperatures and actual microclimatic temperatures recorded in potential habitats of biting midges (*Culicoides*) and mosquitoes (*Anopheles*, *Culex* and *Aedes*); 2) compare the seasonal impacts of microclimatic and meteorological temperatures on the development time of bluetongue, Schmallenberg, West Nile and dengue virus, vivax malaria and Dirofilaria parasites and blood meal digestion period of biting midges and mosquitoes; 3) develop a model to predict the hourly microclimatic temperatures of a habitat, based on available parameters from meteorological institutes.

## Results

### Microclimatic vs. meteorological temperature

We observed large variations between temperatures recorded at six microclimatic habitats at three different heights, and the closest DMI weather station (Table 1). Although there were relatively small differences in the average monthly mean temperatures between DMI records and the different microclimatic habitats (for example 1.0 to 1.3 °C in dry meadow in May 2015), the variation in temperature at specific times was high (for example, −5.8 to 11.4 °C in dry meadow in May 2015), as reflected in hourly differences (5% and 95% percentiles) of microclimatic temperature (Table 1). Overall, the horse and cattle fields had higher monthly mean temperatures (range: 0.01 & 3.2 °C) at all heights (10 to 110 cm) during the entire season (June to October). The upper and mid-height of dry meadow and hedge habitats had higher monthly mean temperatures (range: 0.30 & 1.6 °C) compared to weather stations (except for October). Forest/trees had similar hourly temperatures to DMI records throughout almost the entire study period (maximum difference 0.57 °C). However, the wet meadow was cooler (range −0.07 & −1.8 °C) than meteorological temperatures in late summer and autumn.

**Table 1:** The temperature recorded by the Danish meteorological Institute (DMI) and mean of the difference (5–95% percentiles) between microclimatic data logger temperatures and DMI temperatures at four different habitats in Denmark, May – October 2015.

Locations	Heights	No. of loggers	Mean Temp °C (5–95% percentiles)					
			May	June	July	August	September	October
DMI (Strødam) (5–95% percentiles) (°C)	2.5 m		**10.2** (4.7–14.9)	**13.4** (8.3–19.2)	**16.4** (10.4–24.5)	**17.6** (11.2–23.6)	**13.4** (7.1–17.8)	**9.3** (5.1–12.8)
**Strødam, Denmark, monthly mean of the hourly difference (5–95% percentiles)**								
Dry meadow (°C) – DMI (°C) (5–95% percentiles)	Upper (1.1 m)	6	**1.0** (−2.9–7.2)	**1.2** (−2.9–7.3)	**1.5** (−3.6–8.6)	**1.3** (−3.9–9.1)	**0.60** (−3.7–8.8)	**0.35** (−3.8–5.8)
	Mid-height (0.55 m)	6	**1.3** (−3.5–8.5)	**1.6** (−5.0–9.2)	**1.6** (−5.7–10.8)	**0.80** (−5.5–9.7)	**0.30** (−6.2–7.4)	**–0.90** (− 5.7–4.1)
	Lower (10 cm)	6	**1.1** (−5.8–11.4)	**1.5** (−5.7–11.8)	**0.85** (−5.7–10.8)	–**0.10** (−5.8–9.8)	**–1.1** (−6.6–6.0)	**–1.4** (−6.1–2.0)
Hedges (°C) –DMI (°C) (5–95% percentiles)	Upper (3.3 m)	6	**1.2** (−2.3–7.0)	**1.7** (−2.3–8.7)	**1.6** (−2.1–8.0)	**0.90** (−2.9–6.5)	**0.35** (−2.6–5.0)	**–0.35** (−2.6–2.6)
	Mid-height (2.2 m)	6	**1.2** (−2.2–7.2)	**1.4** (−2.2–7.8)	**1.2** (−2.3–7.2)	**0.75** (−2.6–5.3)	**0.20** (−2.7–4.8)	**–0.40** (−2.6–1.9)
	Lower (10 cm)	6	**−0.15** (−3.7–4.7)	**−0.75** (−3.9–2.4)	**−1.2** (−4.2–1.5)	**−1.3** (−4.5–1.9)	**−1.5** (−5.1–1.0)	**−1.5** (−4.8–0.6)
Tree (°C) –DMI (°C) (5–95% percentiles)	Upper (9.4 m)	2	**0.10** (−2.2–2.4)	**0.12** (−2.3–2.4)	**−0.02** (−2.9–2.3)	**−0.06** (−3.0–2.6)	**0.10** (−2.5–2.5)	**0.07** (−1.7–2.6)
	Mid-height (6.8 m)	2	**0.30** (−2.2–3.0)	**−0.01** (−2.5–2.2)	**−0.13** (−3.6–2.2)	**−0.16** (−3.1–2.3)	**−0.15** (−2.7–2.2)	**0.57** (−0.3–2.0)
	Lower (3.0)	2	**0.47** (−2.1–3.3)	**0.01** (−2.7–2.2)	**−0.15** (−3.6–2.1)	**−0.20** (−3.3–2.4)	**−0.19** (−2.7–2.2)	**0.06** (−1.8–2.4)
Wet meadow (°C) – DMI (°C) (5–95% percentiles)	Upper (0.55 m)	6	**0.18** (−3.5–5.1)	**0.04** (−3.2–3.2)	**−0.65** (−4.1–20.0)	**−1.5** (−4.8–1.0)	**−1.2** (−4.3–1.0)	**−1.16** (−3.4–0.6)
	Lower (0.1 m)	6	**0.42** (−3.3–5.8)	**−0.07** (−3.7–3.2)	**−0.77** (−4.6–2.3)	**−1.8** (−5.2–1.0)	**−1.3** (−4.3–1.1)	**−1.1** (−3.3–0.7)
**Faxe, Denmark, (5–95% percentiles)**								
DMI (Faxe) (5–95% percentiles) (°C)	2.5 m		**10.0** 5.3–14.1)	**13.1** (7.3–18.8)	**16.1** (10.3–22.7)	**17.6** (11.5–22.8)	**13.3** (7.5–17.2)	**9.3** (5.8–13.3)
**Faxe, Denmark, monthly mean of the hourly difference (5–95% percentiles)**								
Cattle Field (°C) – DMI (°C) (5–95% percentiles)	Upper (1.2 m)	2	**NA**	**1.9** (−1.8–7.6)	**2.3** (−1.7–8.5)	**1.4** (−2.4–7.1)	**1.2** (−2.3–7.3)	**0.30** (−2.3–3.9)
	Mid-height (0.60 m)	2	**NA**	**2.3** (−1.5–7.9)	**2.3** (−1.4–8.2)	**1.3** (−2.2–6.6)	**1.8** (−2.1–9.3)	**0.86** (−1.9–5.5)
	Lower (0.10)	2	**NA**	**1.2** (−2.0–4.6)	**0.64** (−2.6–4.3)	**0.38** (−3.5–6.2)	**1.3** (−3.5–10.5)	**0.01** (−3.2–3.2)
Horse Field (°C) – DMI (°C) (5–95% percentiles)	Upper (1.2 m)	2	**NA**	**2.3** (−1.2–7.5)	**2.5** (−1.3–8.3)	**1.02** (−3.0–6.2)	**1.3** (−2.2–9.6)	**0.64** (−1.8–7.1)
	Mid-height (0.60 m)	2	**NA**	**2.5** (−1.4–8.1)	**2.9** (−1.5–11.0)	**1.4** (−3.1–6.6)	**1.5** (−2.4–10.9)	**0.38** (−2.1–5.4)
	Lower (0.10)	2	**NA**	**3.2** (−1.5–10.9)	**2.9** (−2.3–11.2)	**2.1** (−4.1–12.5)	**1.1** (−3.0–9.4)	**0.10** (−2.9–4.9)

Data from cattle and horse fields were available from 4^th^ June - 31^st^ October 2015, whereas data from other habitats were available from 1^st^ May–31^st^ October 2015.

**Figure 1:**
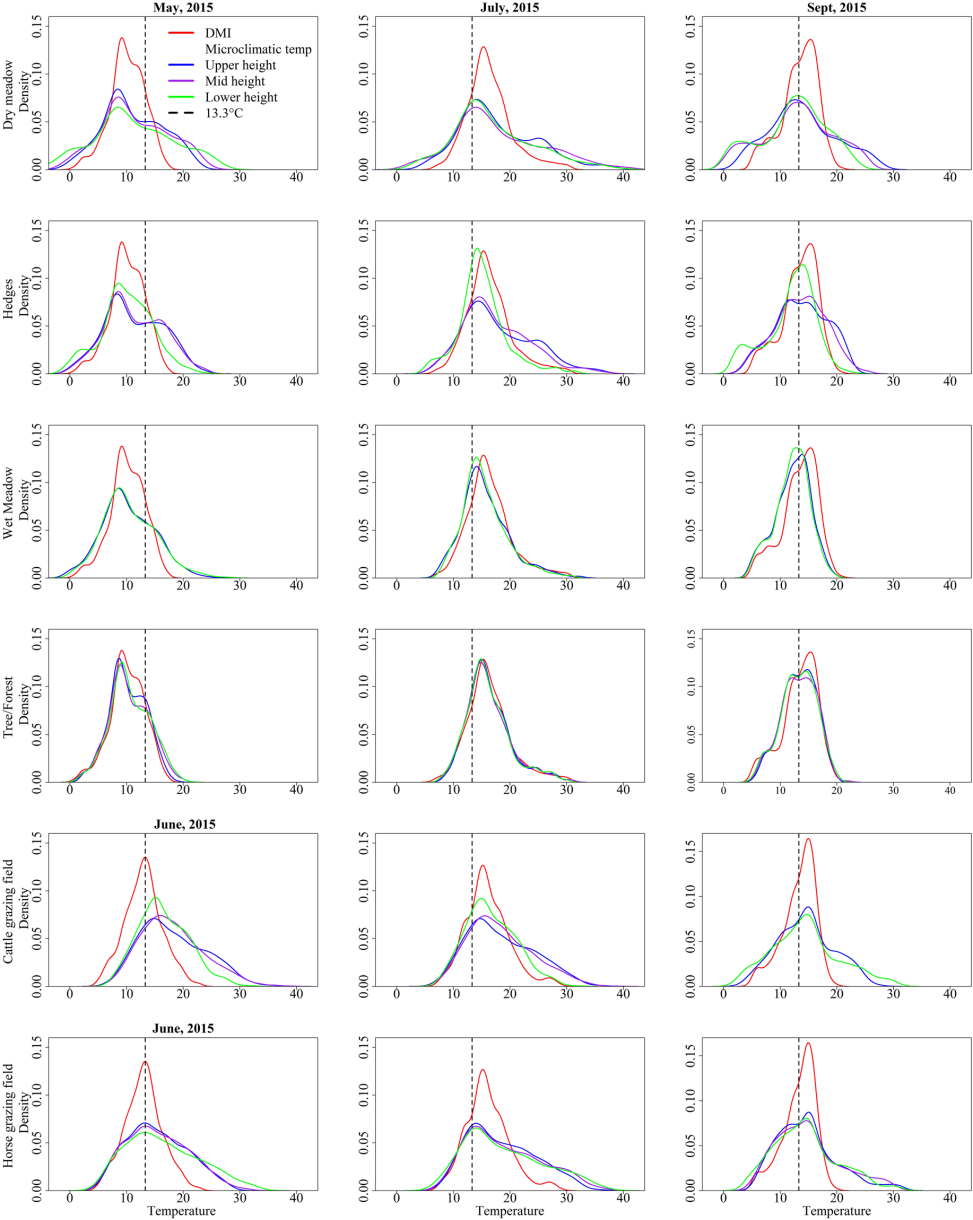
Hourly temperatures measured at six different microclimatic habitats and from the weather stations of the Danish Meteorological Institute (DMI). For illustrative purposes, only the months May, (June, for horse and cattle fields), July and September are depicted. The vertical (black dashed) line is the threshold temperature for pathogen development (Schmallenberg virus, 13.3 °C). Data from cattle and horse fields were available from 4^th^ June - 31^st^ October 2015, whereas data from other habitats were available from 1^st^ May –31^st^ October 2015. The temperature variation was considerably higher in microclimatic habitats, and differences were more evident in spring (May or June) and autumn (September). The dry meadow, horse field and cattle field were warmer than other habitats.

We generated frequency distributions of hourly temperatures of microclimatic habitats and weather stations (Fig. 1) and found large variations in the microclimatic habitats, which were both warmer and colder than the temperatures from DMI. We compared the number of hours during which temperatures were above 13.3 °C (the threshold temperature for Schmallenberg virus replication in *Culicoides* ^5^) in the microclimatic habitats and DMI records, and found that the microclimatic habitats had experienced more hours with temperatures above 13.3 °C (p < 0.05), except in wet meadow (July to October; Fig. 1). The microclimatic habitats (especially dry meadow, hedges and cattle and horse fields) were above the threshold temperature for many hours in spring and autumn. In contrast, the wet meadow had fewer hours above 13.3 °C (Fig. 1).

**Figure 2:**
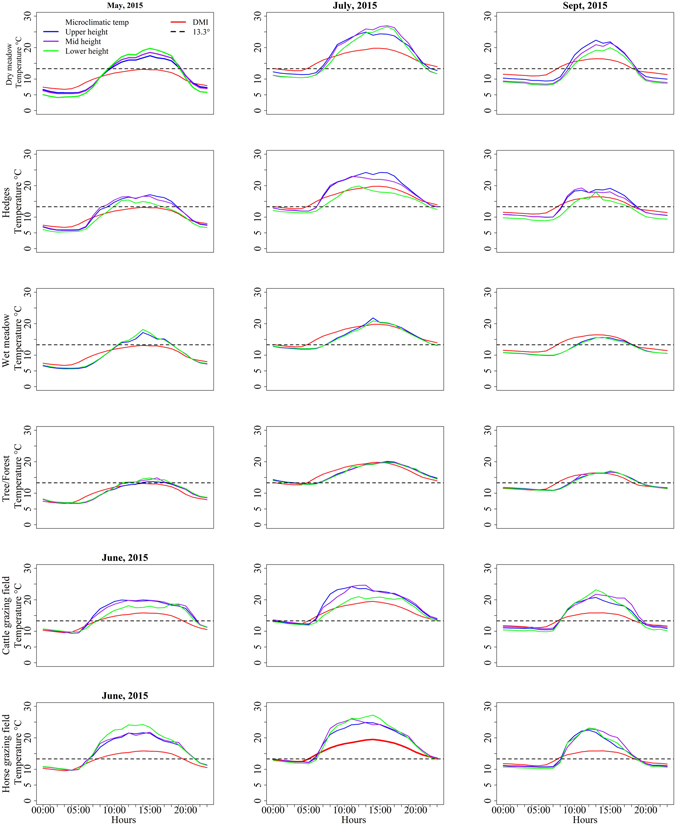
The daily temperature variation at different microclimatic habitat heights and from the weather stations of the Danish Meteorological Institute (DMI). For illustrative purposes, only the months May, June (for the horse and cattle fields), July and September are depicted, with the y-axis showing the average temperature for the month at the time of day given on the x-axis. The horizontal (black dashed) line is the threshold temperature for pathogen development (Schmallenberg virus, 13.3 °C). Data from cattle and horse fields were available from 4^th^ June - 31^st^ October 2015, whereas data from other habitats were available from 1^st^ May – 31^st^ October 2015. Microclimatic habitats were cold at night and warm during the day, and the difference between microclimatic temperatures and DMI temperatures was more evident in the spring (May or June) and autumn (September). The dry meadow, horse field and cattle field were warmer than other habitats.

Microclimatic habitats were on average 3.5 to 5 °C warmer than DMI temperatures during midday, and 1 to 3 °C cooler during midnight (Fig. 2). In May, dry meadow (upper height) had mean hourly temperatures >13.3 °C for 10 hours during the day, whereas DMI temperatures did not record a single hour of the day above that temperature (p < 0.001; Fig. 2). During spring (May to June), the lower height of the microclimatic habitats had high temperatures during the day and low temperatures during night compared to the upper and mid-height of the habitats. During summer and autumn, the upper height of the habitats had high temperatures compared to the lower height. The difference in hourly temperature between the upper and lower height of the habitats in dry meadow was: −0.10 °C in May, −0.26 °C in June, 0.66 °C in July, 1.4 °C in August, 1.6 °C in September and 1.1 °C in October (Table 1). The temperatures of the upper height of dry meadow were 3 to 5 °C higher during the day when solar radiation was >100 W/m^2^, and 1 to 2.5 °C lower during the night when solar radiation was zero, compared to the DMI temperatures. Similar patterns were also observed in other microclimatic habitats.

### Extrinsic incubation period (EIP)

**Figure 3:**
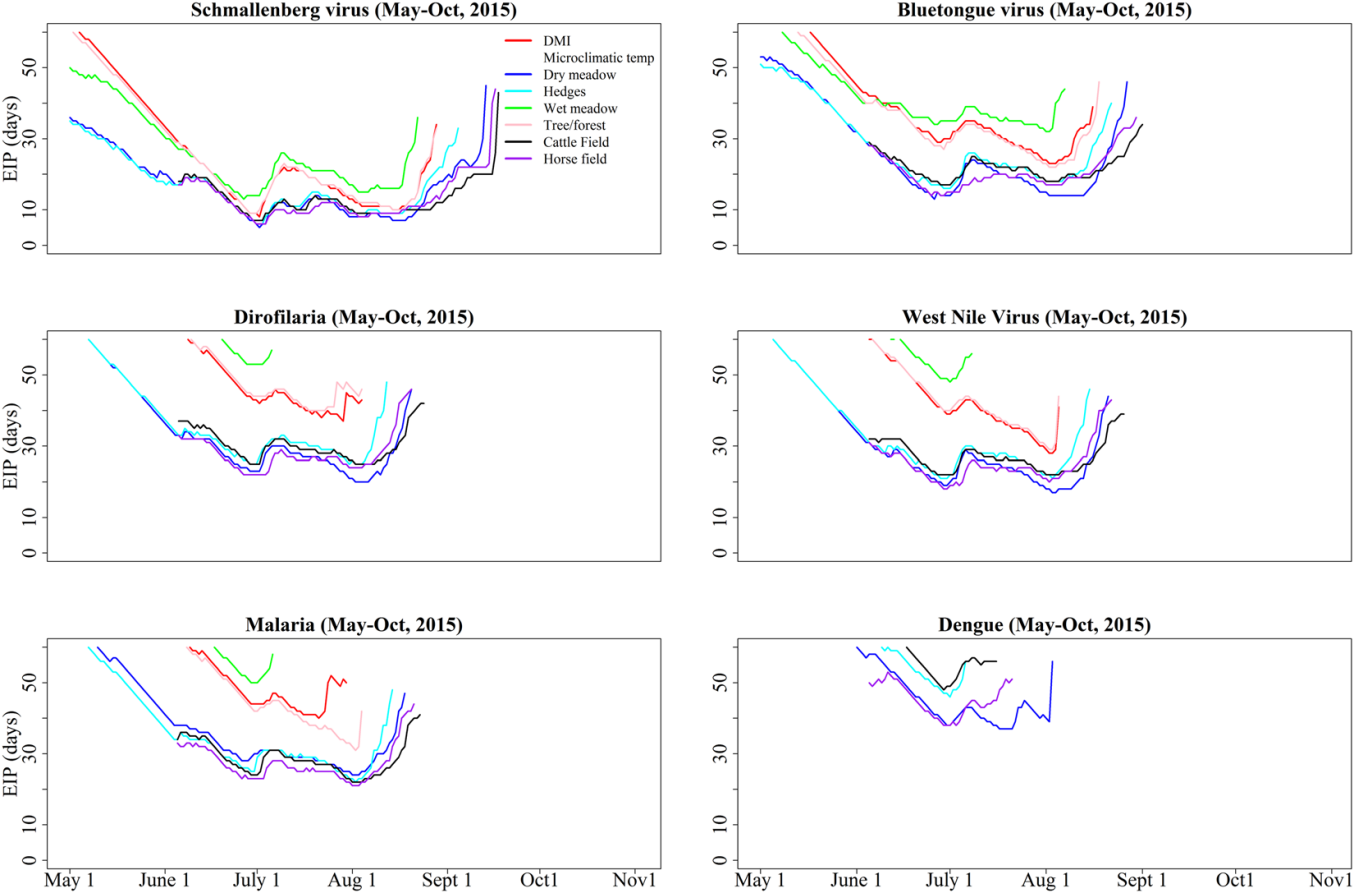
The extrinsic incubation period (EIP) of bluetongue, Schmallenberg, dengue and West Nile virus, Dirofilaria and *Plasmodium sp* (malaria) calculated from temperatures in different microclimatic habitats and from the Danish Meteorological Institution (DMI), May - October 2015, Denmark. Data from cattle and horse fields were available from 4^th^ June - 31^st^ October 2015, whereas data from other habitats were available from 1^st^ May – 31^st^ October 2015. An extended period and faster virus development were observed at microclimatic habitats than for DMI temperatures. Dengue virus did not develop fully when modelled with DMI data.

Different pathogens have different EIP durations in different microclimatic habitats (Fig. 3). Overall, all the pathogens showed faster development using the microclimatic temperatures in dry meadow, hedges, and cattle and horse fields compared to DMI temperatures. Schmallenberg virus, for example, could complete development at a mean of 24.4 days with DMI temperatures, 13.3 days with cattle field temperatures, 13.4 days with horse field temperatures, 15.2 days with dry meadow temperatures, 15.7 days with hedge temperatures, 24.1 days with forest/tree temperatures and 25.5 days with wet meadow temperatures between 4^th^ May and 21^st^ August (the time period during which all habitats allowed for viruses to develop fully). For most of the pathogens, the microclimatic habitats allowed for a longer transmission season compared to DMI temperatures (Fig. 3). For example, the model using DMI data showed that bluetongue virus could only successfully develop in vectors for 92 days after infection, from 17^th^ May to 16^th^ August, with a mean of 36 days. For microclimatic temperatures in the upper height of dry meadow habitat, virus development was possible for 117 days starting from 1^st^ May to 27^th^ August, with a mean of 27 days. Between 4^th^ May and 21^st^ August (when all habitat temperatures facilitated the development of bluetongue virus) the minimum EIP for DMI data was 23 days, compared to 13 days for dry meadow temperatures (Fig. 3).

### The blood meal digestion period

**Figure 4:**
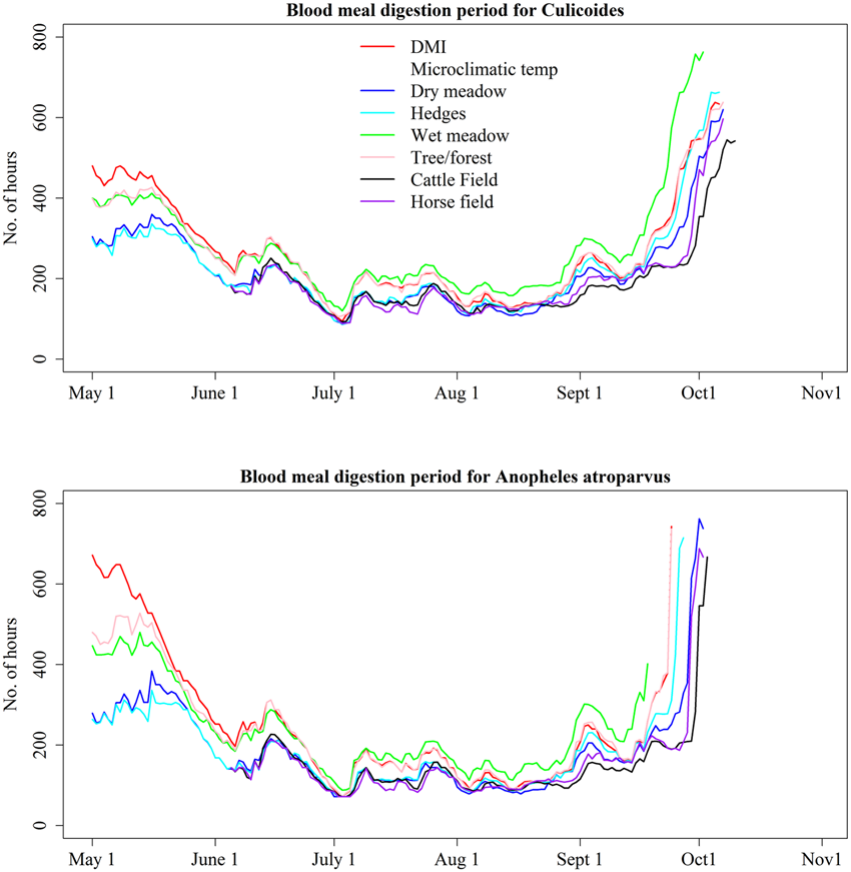
The blood meal digestion period of biting midges (*Culicoides*) and mosquitoes (*Anopheles atroparvus*) calculated from temperatures in different microclimatic habitats and from the Danish Meteorological Institution (DMI), May – October 2015, Denmark. Data from cattle and horse fields were available from 4^th^ June – 31^st^ October 2015, whereas data from other habitats were available from 1^st^ May – 31^st^ October 2015. A maximum of 60 days was assumed for the blood meal digestion in *Culicoides* and mosquitoes. The blood meal digestion was faster in microclimatic habitats than DMI data, and the difference was more evident in the spring (May) and autumn (October).

The average number of hours required for complete digestion of a blood meal in biting midges was 258 hours with DMI temperatures, 207 hours with dry meadow, 214 hours with hedges, 280 hours with wet meadow and 252 hours with forest/tree temperatures (1^st^ May to 2^nd^ October). It took on average 258 hours for *Anopheles* mosquitoes to complete blood meal digestion for the DMI temperatures, 167 hours for dry meadow, 169 hours for hedges, 235 hours for wet meadow and 224 hours for forest/tree temperatures (1^st^ May to 18^th^ September; Fig. 4).

### The microclimatic temperature model

We performed multiple linear regressions to express the microclimatic temperature of the six different habitats using standard climatic variables from DMI. The six models were able to explain 87 to 96% of the variation in the habitats (Table S1), where the DMI temperature, the temperature of the previous hour, solar radiation, wind speed, humidity, precipitation, months and hours of the day were all important variables in predicting the hourly microclimatic temperature (Figure S2). We evaluated these key drivers of the model in order to understand the role they played in predicting microclimatic temperature. When exploring a variable, we kept all other variables fixed at the median value, and tested the minimum, first quantile, median, third quantile and maximum values of the variable in question. These evaluations showed that microclimatic temperatures increased with increasing solar radiation. For example, on a sunny day, compared to DMI temperature, microclimate habitats were 0.5 to 3.5 °C warmer when solar radiation was 200 (W/m^2^), and 4 to 10.5 °C higher when solar radiation was 800 (W/m^2^) (Figure S2). High values of precipitation and humidity negatively affected the microclimatic temperature (with the exception of precipitation in hedges). As the precipitation and humidity increased, the difference between microclimatic and DMI temperature decreased. Wind speed played different roles in the different microclimatic habitats (Table S1). For example, the microclimatic temperature in forest/trees and cattle fields dropped as the wind speed increased, yet in other habitats, the wind speed seemed to marginally increase the microclimatic temperature. Validation of the model is described in supplementary document S3 (Model Validation).

## Discussion

Despite the cool Scandinavian climate in Denmark, there have been outbreaks of vector-borne diseases in recent times^24^. Microclimatic temperatures may help us understand and quantify the potential for transmission of vector-borne diseases in Northern Europe. We found only a modest discrepancy between the daily mean temperatures from the national meteorological institute data and our recordings in six microhabitats. We did, however, identify a much larger daily variation in microclimatic temperatures compared to national meteorological temperatures, which resulted in higher maximum temperatures at most microclimatic habitats.

The microclimatic habitats (particularly the dry meadow, hedges and cattle and horse fields) were generally warmer than DMI temperatures - possibly due to the direct exposure to sunlight. The agreement between forest/tree temperatures and DMI temperatures may be due to the similar height of the microclimatic temperature loggers and the DMI weather stations, or because of the high exposure to wind. The lack of direct sunlight in wet meadow habitats resulted in a cooler microclimate than the DMI temperatures. Seasonally, temperature variation differed considerably at different heights above the ground, probably due to the growth of grass vegetation during the summer. During the early period of the warm season (spring), the lower heights of the habitats were warmer than the upper and mid-height whereas during late summer and autumn, the temperature at the lower habitats became cooler as the vegetation grew and blocked the sunlight. Microclimatic temperatures are therefore complex and very dependent on local factors such as seasonal vegetation growth.

A number of studies are available on microclimatic temperatures, but most of them concentrated on habitats different from those in our study^9–11, 13, 15^. Examples include forest microclimates^9, 12^, underground temperatures^9, 10^, sea temperatures^14^, mosquito breeding habitats (water)^11^ and tropical climates^16^ – none of which are comparable to our study. One study in tropical settings in Chennai, India, found microclimatic habitats were warmer at night, in contrast to our findings^16^. The study in Chennai was conducted in densely packed urban environments described as ‘urban heat islands’, which is in stark contrast to our rural temperature data collection sites^25^. Another study looked at seasonal variation in microclimatic conditions in domestic and peri-domestic habitats in rural Argentina and found that microhabitats were generally 5.0 to 5.6 °C higher compared to the ambient temperature^17^. However, we found that the difference between microclimatic and meteorological temperature was mostly in the hourly distributions and only to a lesser extent in daily mean or median temperatures, since microclimatic habitats become warmer during the day and cooler during the night than the meteorological temperature. Temperatures below the threshold have no impact on virus development or the blood meal digestion period. Therefore, daily or monthly mean temperature could mask the hourly variations of temperatures in the model and risk either underestimation (when the mean temperature is below threshold) or over-estimation (when the mean temperature is above threshold) of virus development and blood meal digestion period. Since microclimatic habitats had more hours above the threshold temperature, the impact of microclimatic temperature on the virus development rate and blood meal digestion was noticeable. Microclimatic temperature is therefore very important, especially for countries like Denmark, where daily and monthly average temperatures are often close to the pathogen-development threshold (10 to 15 °C).

Based on our EIP estimates for vector-borne pathogens of the year 2015, we found two important differences between models using meteorological and microclimatic temperatures for estimating the transmission intensity: 1) pathogens develop at a faster rate in microclimatic habitats (except wet meadow) and 2) there is a longer season for vector-borne disease transmission when modelling with microclimatic temperatures compared to meteorological institute temperatures. The increased duration of the pathogen-development season in the vector is an important finding. Using the microclimatic temperatures of 2015, we found that complete virus development was possible in a period of at least 2.5 months for dengue, 4 months for malaria, 4.5 months for Dirofilaria and West Nile Virus, and 5 months for bluetongue and Schmallenberg virus. In recent years, Schmallenberg virus has been detected in late autumn (September) in Denmark and other European countries^26^. When using meteorological temperature in our transmission model, the Schmallenberg virus did not develop after mid-August in 2015. However, when the observed microclimatic temperature was used in the same model, virus development was possible for one additional month. Therefore, the microclimatic temperature increased both the daily transmission rates and the number of days where transmission could take place, potentially leading to a dramatic change in the accumulated number of cases at the end of the season with an outbreak starting early in summer. We started collecting microclimatic data from May (in dry meadow, wet meadow, hedges, and forest/trees) and June (in cattle and horse fields), but our model shows that virus development and blood meal digestion might occur even earlier in the year. However, it is important to note that although virus development rate is faster at higher temperatures, the vector survival rate is correspondingly lower.

While standard temperatures are available from national meteorological offices for any site in Europe, hourly microclimatic temperatures are not. Our models enabled us to predict the temperature of different microclimatic habitats based on available standard variables from a meteorological institute. The important meteorological variables in our model (i.e. meteorological temperature, solar radiation, humidity, precipitations and wind speed) have also been identified as key variables for predicting microclimatic temperature in previous studies^10, 13^.

The temperature during the previous hour also played an important role in predicting microclimatic temperature, indicating that it takes time for the temperature in a microclimatic habitat to change. For example, if solar radiation heats up the vegetation and soil in the habitats, it will take time to cool down, while the standard meteorological temperature 2 m above ground can change much more rapidly. In addition, a correlation was found between wind speed and solar radiation. During the day when solar radiation was higher, the wind speed increased, and the opposite was observed at night. We found relative humidity was more important than precipitation for predicting microclimatic temperature (see Table S1), though this might be because there were only a few days with rain during our study period. Our models included the month of the study period as a categorical variable, and predicted microclimatic temperatures therefore differed from month to month, despite all other input variables being the same. This suggests that the models are only useful for habitats and climates that resemble the Danish pattern of vegetation growth and climate seasonality (e.g. northern Europe and southern Scandinavia). In general, the model was good at predicting the microclimatic temperature, but the predictions underestimated the temperature compared to observed microclimatic temperature when there was heavy rain. Wind speed in the dry meadow, hedges and horse field were positively correlated with microclimatic temperature. We selected dry meadow, wet meadow and hedges from a natural reserve area encircled by large trees, so wind speed was obstructed by forest and could not change the temperature at the lower and mid-height of dry meadow and hedges habitats. Likewise, the horse fields were also surrounded by trees, and the influence of wind speed might be different in other microhabitats not surrounded by forest.

Our EIP estimates of different pathogens are supported by empirical findings in Denmark. Our estimate shows that bluetongue and Schmallenberg virus transmission is possible in even late autumn. The country experienced bluetongue virus outbreaks in 2007 and 2008^24^ and Schmallenberg virus outbreaks in the autumn, 2012^27^. There was a number of outbreaks of malaria in the sixteenth to eighteenth century and as recent as in the nineteenth century^28^, with 33 cases reported as late as 1911 in Denmark^29^, which supports our findings that the microclimatic temperature was warm enough to allow malarial parasite development in mosquitoes at that time. The vectors for West Nile virus and Dirofilaria have recently been reported in Denmark^30^. Our findings suggest that it is likely that Dirofilaria (minimum EIP- 20 days) and West Nile virus (minimum EIP- 17 days) could be transmitted in Denmark if introduced. No known vectors for dengue are currently reported in Denmark, and based on our estimation, if they are introduced and become infected; they will have to survive a minimum of 45 days to be able to transmit the virus given the observed temperature in 2015 in Denmark.

The resting sites for biting midges and mosquitoes are largely unknown, but different types of mosquitoes and biting midges might rest on different types and heights of vegetation^31–33^. The distribution of mosquitoes and *Culicoides* depends on the distribution of their hosts and the microclimate of their resting habitats^34^. The resting sites of adult *Culicoides* were reported to be dense vegetation, leaf sheaths and bushes^35^. Carpenter (1951) reported adult *Culicoides* resting on ground litter and on the underside of foliage; he found the equally distributed adult *Culicoides* at 7, 23 and 35 feet above ground^36^. Carpenter *et al*. (2008) confirmed the presence of *Culicoides impunctatus* in extremely large numbers on European white birch (*Betula pubescens*), a deciduous tree native and abundant throughout northern Europe^37^. Natural resting sites for mosquitoes of the eight genera including *Anopheles*, *Culex* and *Aedes* were reported by Burkett-Cadena *et al*. (2008). The predominantly natural resting sites of the adult female mosquitoes were small and large tree cavities, understory vegetation, and trash cans^38^. Above 35 °C all *Anopheles* seek even darker and consequently cooler shelter. They also avoid habitats of high temperatures with low humidities^39^. The literature suggests that both mosquitoes and *Culicoides* do not select resting sites randomly, rather they seek favorable microhabitats to rest in and these microhabitats may be related to shade and increased humidity. However, little is known about their resting behavior during the low temperatures experienced in Scandinavia during spring and autumn when *Culicoides* abundance can be high. Biting midges and mosquitoes can quickly move between microhabitats on a farm and may move between resting sites to optimize conditions. We here quantified a range of microclimates related to farms and grazing areas, but there is a need to understand the vector’s actual selection of resting microhabitats in order to model pathogen development times and blood meal digestion period in the vectors and to ultimately model transmission potential of these vectors.

We collected microclimatic temperatures from different habitats. Although we do not know where the vectors rest, we believe the range of temperatures obtained from microclimatic habitats at different heights are more relevant for disease transmission than temperatures measured in a box 2 m above ground by a standard weather station. We do not know which particular microclimatic temperature is most relevant to select as resting site for the different vector species, so instead of single EIP estimates based on a specific microclimate, we suggest the EIP and blood meal digestion period are better estimated as a range based on different microclimates within a given area of interest. If microclimatic temperatures are not available or not convenient to collect, we recommend using the models described here to predict temperatures, providing the settings are similar to the Danish climate.

Successful completion of EIP of a vector-borne disease depends on the life span of the insect. In our model, we considered a life-span window of 60 days for mosquitoes and biting midges. In published literature, the life span of mosquitoes has been described as 24–67 days^40^ and for biting midges between 10 to 30 days, but they can survive up to 90 days under very cool weather conditions^41^.

## Conclusion

Our model shows that viruses develop at a faster rate and have a longer season of transmission when modelled with microclimatic temperature compared to meteorological temperature. The reason for this is that microclimatic temperature differs greatly from meteorological temperature. Microclimatic habitats were warmer during the day and cooler during the night compared to the meteorological temperature, despite relatively small difference in the mean temperatures of both estimates. Using standard meteorological temperatures (especially in a cool temperate climate like in Denmark) can substantially underestimate the potential for vector-borne disease transmission, particularly for zoonotic pathogens like Dirofilaria and West Nile virus, and human pathogens like malaria and dengue. Using daily or monthly mean temperature carries a risk of underestimation or overestimation of vector-borne disease transmission parameters. Although no simple relationship exists between microclimatic temperature and meteorological temperature, it is possible to model a range of microclimatic temperatures based on standard meteorological data and use this range of estimated temperatures to drive vector-borne disease transmission models.

## Methods

### Study sites

We collected microclimatic and meteorological temperatures at Strødam and Faxe in Denmark. Strødam (N55.96007°, E012.27465°) is a 20-hectare natural reserve area with protected woodlands, meadows and open fields. We chose four nearby habitats with different vegetation types: dry meadow, wet meadow, hedges and woodland (forest/trees) at which to record the microclimatic temperature. We set up a portable weather station to record the temperature and other parameters from the same area in order to identify the important variables for modelling microclimatic temperature that should be requested from DMI. Faxe is a small municipality south of Copenhagen. We recorded the microclimatic temperature from one horse grazing field (N 55.22383°, E012.04494°) and one cattle grazing field (N 55.20258, E012.11440) in the municipality.

### Recording Microclimatic temperature

Microclimatic temperatures were recorded using “Temperature logger (21G) ^(TM)^” data loggers that record the temperature in a single spot of about 3 square centimeters^42^. The temperature loggers are portable battery-powered instruments that can be deployed anywhere to record the temperature of the surroundings^42^. We recorded temperatures at three different heights from dry meadow, hedges, forest/trees, cattle and horse field, but the vegetation in the wet meadow was only tall enough to allow us to measure at two different heights in this habitat. Dry meadow is grassland where green succulent grass grows during spring, summer and early fall. The vegetation grows up to 1.3–1.5 meters. Wet meadow is a wetland where standing water could be present for a short period of time during the growing period. The vegetation grows up to 0.50–0.75 meters. Hedges are collections of plants (usually shrubs) and other tree species implanted in a way to create a barrier or to mark a boundary usually between two neighboring areas. The shrubs grow up to 3.0–3.5 meters. For forest/tree, we chose a tall deciduous tree (*Prunus sp*) approximately 9.5 meters high in a forest in Strødam. The tree was located deep in the forest surrounded by similar trees. Cattle grazing land were chosen from grazing land near a wheat field in Faxe, Denmark. We recorded the temperature from the grassland close to the electric fence. The grasses grow up to 1.0–1.5 meters. Horse grazing land was chosen from a place where horses were allowed to graze in a field close to a horse stable at Faxe, Denmark. This grassland habitat resembles dry meadow but was surrounded by large trees. We used three different sites for each habitat (except forest/trees, cattle and horse field). For the dry meadow, wet meadow, hedges, cattle and horse field, the temperature loggers were placed from 10 cm above the ground (lower) to the top of the vegetation: 1.1 m for dry meadow, cattle field and horse field, 3.3 m for hedges, and 0.60 m for wet meadow. The mid-height loggers were placed approximately halfway between the upper and lower loggers. In forest/trees, we placed the loggers at 9.4 m (upper), 6.8 m (mid) and 3.0 m (lower). Two temperature loggers were placed in one small transparent plastic bag at each height after removing the air manually. The small plastic bag was used to protect the loggers from being directly exposed to rain. The loggers recorded the temperature every 30 minutes and we downloaded the temperatures to a portable data recorder “TempTec-R”^43^ at 15–30-day intervals. In total, there were 54 loggers (18 in dry meadow, 18 in hedges, 12 in wet meadow and 6 in trees) in Strødam and 12 loggers (6 in the horse field and 6 in the cattle field) in Faxe.

### Meteorological climatic data collection

Based on our initial analysis from the portable weather station data from Strødam, we identified hourly temperature (° C), solar radiation (W/m^2^), humidity (relative humidity, in %), wind speed (m/s, 10 minutes average), and precipitation (mm/hour) as potentially important variables for predicting microclimatic temperature. We then requested those data from DMI from 1^st^ May to 31^st^ October 2015. We received the meteorological data recorded by weather station at Sjælsmark (N 55.94407, E 12.27065), 1.8 km away from the Strødam data collection point, and at Faxe Ladeplads (N55.2076, E 12.09807), 1.3 km away from the cattle field and 4.6 km away from our horse field.

### Data analysis

We calculated summary statistics of temperatures recorded from DMI expressed as the mean and 5% and 95% percentiles for each month (May to October 2016; Table 1). We then deducted the hourly DMI temperature from the hourly microclimatic temperature for each height for all six months and reported their mean and 5 and 95% percentiles. Then, we plotted the number of hours of microclimatic and DMI temperature data from each month and habitat (Fig. 1). We plotted the daily variation in temperature at different microclimatic habitat heights and DMI for each month by calculating the monthly average, and then comparing the number of hours ≥ 13.3 °C to the DMI temperature (Fig. 2). The summary statistics and graphs were prepared in the statistical software R^44^.

### Linear regression to predict microclimatic temperature

We extracted hourly temperature, solar radiation, wind speed, precipitation and humidity information from the DMI weather data. These variables were all included in the initial model to express the microclimatic temperature of a particular habitat. In addition, the month and time of day (as categorical variables), and the height of the data loggers were included in the linear regression model. The following steps were then taken:

1. The initial model included eight variables: meteorological temperature, solar radiation, wind speed, precipitation, humidity, time of day (24 hours), month (each month between May and October as a categorical variable) and height of the habitats (as a categorical variable).
2. The day also played a role in our microclimatic models, but it was not practical to consider each day as a categorical variable. The temperature on 31^st^ May is more likely to be influenced by both May and June than just May, so we used a floating weight for each day, giving the 15^th^ day of each month (taken to be the mid-point) a weight of 1, with other days influenced by neighbouring months, as expressed by the formula:
  $${\rm{Month}}({\rm{x}})=15-({\rm{absolute}}({\rm{Date}})/{\rm{No}}{\rm{.}}\,{\rm{of}}\,{\rm{days}}\,{\rm{of}}\,{\rm{month}})$$
  This is a 30 or 31-day window and the sum of the weight of each day is always 1. To avoid negative values originating beyond these 30 or 31days, all negative weights were set to 0 (Figure S5).
3. Five significant interactions were identified in the initial analysis and were added to the model:
  - Solar radiation and wind speed
  - Rain and humidity
  - Solar radiation and month
  - Wind speed and height
  - Solar radiation and height
4. We added a 1-hour lag of DMI temperature (Temp_DMI(t-1)_) in the model
5. The final model was:

$$\begin{array}{rcl}({{\rm{Temp}}}_{{\rm{micro}}}) & = & ({{\rm{Temp}}}_{{\rm{DMI}}})+({{\rm{Temp}}}_{\mathrm{DMI}(t-1)})+{\rm{solar}}\,{\rm{radiation}}+{\rm{wind}}\,{\rm{speed}}\\  &  & +\,{\rm{humidity}}+{\rm{Month}}\,{\rm{weight}}\,(\mathrm{May})+{\rm{Month}}\,{\rm{weight}}\,({\rm{June}})\\  &  & +\,{\rm{Month}}\,{\rm{weight}}\,({\rm{July}})+{\rm{Month}}\,{\rm{weight}}\,({\rm{August}})\\  &  & +\,{\rm{Month}}\,{\rm{weight}}\,({\rm{September}})+{\rm{Month}}\,{\rm{weight}}\,({\rm{October}})\\  &  & +\,{\rm{Time}}\,{\rm{of}}\,{\rm{day}}+\,({\rm{solar}}\,{\rm{radiation}}\,\ast \,{\rm{wind}}\,{\rm{speed}})\\  &  & +\,({\rm{precipitation}}\ast {\rm{humidity}})+\,({\rm{solar}}\,{\rm{radiation}}\ast {\rm{month}})\\  &  & +\,({\rm{wind}}\,{\rm{speed}}\ast {\rm{height}})+\,({\rm{solar}}\,{\rm{radiation}}\ast {\rm{height}})\end{array}$$

where Temp_micro_ and Temp_DMI_ are hourly microclimatic and DMI temperatures in Celsius, and height is a categorical value representing the upper, mid-height and lower loggers from the ground.

### Calculating the extrinsic incubation period (EIP) of vector-borne diseases

We calculated the EIP of four viral diseases: bluetongue virus, Schmallenberg virus, West Nile and dengue virus, as well as Dirofilaria and the malaria parasite (*Plasmodium vivax)*, using the same hourly temperature as described for the microclimatic temperature model (Table 2). We assumed zero pathogen development below the threshold temperature for each pathogen^4, 5, 19–21^. In addition, we assumed zero growth ≥34.4 °C for malaria, as suggested by recent studies^6, 16^. This is a rate summation model where virus development is calculated hourly and summed up until the virus development is completed (reached a value of 1)^4–6, 19–21, 45^ or blood meal digestion^4, 46^ is completed. The model was developed in the Statistical Software SAS^47^.

**Table 2:** The equations used to model the changes in the Extrinsic Incubation Period (EIP) of six different pathogens using temperatures from the Danish Meteorological Institute (DMI) and microclimatic temperatures recorded at Strødam and Faxe, May–October 2015.

Pathogens	Equations	References
Bluetongue virus	0.0003 × T × (T−10.4057)/24	4
Schmallenberg virus	(0.019 × (T – 13.3))/24	5
Dengue virus	(−0.1393 + 0.008*T*)/24	21
*Dirofilaria immitis*	(T −14/130)/24	19, 45
Malaria	(0.000126 × T (T–14.244) × √(34.4-T))/24	16
West Nile virus	(−0.132 + 0.0092 × T)/24	20

T = hourly temperature.

### Estimating the blood meal digestion period in Culicoides (biting midges) and mosquitoes

We estimated the time (in hours) required for *Culicoides sp* and *Anopheles atroparvus* to digest a blood meal (Table 3), as described elsewhere^4, 46^, assuming a 60-day life cycle for both *Culicoides* and mosquitoes. This is a rate summation model where blood meal digestion is calculated for each hour and summed up until the blood meal digestion is completed (reached a value of 1).

**Table 3:** The equations used to model the changes in the blood meal digestion period of biting midges (*Culicoides*) and mosquitoes using temperature from the Danish Meteorological Institute (DMI) and microclimatic temperature recorded at Strødam and Faxe, May - October 2015.

Species	Equations	References
*Culicoides sp* (vector of bluetongue and Schmallenberg virus)	(0.0002 × T × (T–3.7) × (41.9–T)^(1/2.71))/24	4
*Anopheles atroparvus* (vector of malaria)	((T − 9.9)/36.5)/24	46

T = hourly temperature.

## Electronic supplementary material

Supplementary Information
