# Supplementary material for: Microclimatic temperatures increase the potential for vector-borne disease transmission in the Scandinavian climate
Source: Sci Rep. 2017 Aug 15;7:8175. doi: 10.1038/s41598-017-08514-9 (PMC5557972; doi:10.1038/s41598-017-08514-9)
Supplement: Supplementary file 1 — Supplementary Information [file 41598_2017_8514_MOESM1_ESM.doc]

**Supplementary documents:**

**Microclimatic temperatures increase the potential for vector-borne disease transmission in the Scandinavian climate**

Najmul Haider^1*^, Carsten Kirkeby^1^, Birgit Kristensen^1^, Lene Jung Kjær^1^, Jens Havskov Sørensen^2^, Rene Bødker^1^

^1^National Veterinary Institute, Technical University of Denmark, Copenhagen, Denmark

^2^Research and Development Department, Danish Meteorological Institute, Copenhagen, Denmark

*=Corresponding author: [najha@vet.dtu.dk](mailto:najha@vet.dtu.dk)

**Supplementary figures and tables**

# **Table S1:** Co-efficients of multiple linear regression analysis of microclimatic temperatures based on parameters collected from the Danish Meteorological Institute (DMI), May - October 2015. Data from cattle and horse fields were available from 4^th^ June - 31^st^ October 2015, whereas data from other habitats were available from 1^st^ May – 31^st^ October 2015.

| Parameters | Dry meadow (ß) | Hedges (ß) | Forest/Trees (ß) | Wet meadow (ß) | Cattle field (ß) | Horse field (ß) |
| --- | --- | --- | --- | --- | --- | --- |
| Intercept | -3.29615 | -1.291392 | 3.142008 | -0.540491 | 7.458548 | -0.567781 |
| DMI temperature | -0.50749 | -0.252880 | -0.218472 | -0.043402 | -0.402979 | -0.222878 |
| Previous hour temp | 1.704965 | 1.256425 | 1.116945 | 0.920504 | 1.315854 | 1.197747 |
| Wind speed | 0.17834 | 0.094738 | -0.062509 | 0.104489 | -0.031547 | 0.194179 |
| Solar radiation | 0.006394 | 0.008497 | 0.000364 | 0.003825 | 0.004614 | 0.010597 |
| Humidity | -0.04047 | -0.013050 | -0.009696 | 0.022252 | -0.036530 | -0.038442 |
| Rain | -2.56966 | -1.421381 | -0.784311 | -1.090726 | -1.262409 | -1.136971 |
| Height (lower) | 1 (ref) | 1 | 1 | 1 | 1 | 1 |
| Height (mid) | 0.424025 | 1.921676 | 0.042642 | NA | 1.175123 | 0.435755 |
| Height (upper) | 1.846005 | 2.016195 | 0.162348 | -0.041653 | 0.884637 | 0.508778 |
| Daily weight (May) | 2.212621 | -0.354158 | -1.194250 | -1.710890 | -1.656993 | 4.395879 |
| Daily weight (June) | 1.51343 | -0.692114 | -1.744299 | -1.589233 | -3.975124 | 3.008218 |
| Daily weight (July) | 4.281054 | -0.050915 | -2.108724 | -0.577108 | -1.932351 | 6.222419 |
| Daily weight (Aug) | 4.403061 | -0.134437 | -2.440829 | -1.300479 | -3.699297 | 4.412191 |
| Daily weight (Sept) | 4.250463 | -0.343676 | -2.445684 | -1.046149 | -1.726114 | 5.295510 |
| Daily weight (Oct) | 4.41985 | -0.746235 | -2.395448 | -2.896761 | -4.420269 | 5.036010 |
| Month (May) | 1 | 1 | 1 | 1 | NA | NA |
| Month (June) | -0.83561 | -0.258953 | 1.016326 | 0.414985 | 1 | 1 |
| Month (July) | -3.52574 | -0.218181 | 1.602317 | -0.011944 | -1.683031 | -2.104695 |
| Month (Aug) | -4.23109 | -0.557553 | 1.634379 | 0.007538 | -0.875904 | -1.639220 |
| Month (Sept) | -3.38687 | -0.301393 | 1.529616 | 0.164828 | -3.146251 | -1.925644 |
| Month (Oct) | -2.41106 | 0.426661 | 1.187070 | 1.042442 | -0.297970 | -1.777821 |
| Time (00:00) | 1 | 1 | 1 |  | 1 | 1 |
| Time (01:00) | 0.250053 | 0.109278 | -0.027686 | 0.023701 | 0.055659 | 0.077398 |
| Time (02:00) | 0.345663 | 0.137103 | -0.064083 | 0.026198 | -0.017128 | 0.094201 |
| Time (03:00) | 0.538556 | 0.244270 | -0.036496 | 0.075849 | 0.033391 | 0.126813 |
| Time (04:00) | 0.545741 | 0.135197 | -0.060453 | 0.029657 | 0.090918 | 0.074449 |
| Time (05:00) | 0.55761 | -0.005559 | -0.068613 | -0.001704 | 0.045653 | -0.175052 |
| Time (06:00) | 0.077547 | -0.386145 | -0.335666 | -0.236922 | 0.082368 | -0.542660 |
| Time (07:00) | -0.42283 | -0.012428 | -0.641177 | -0.409374 | 0.524860 | -0.417640 |
| Time (08:00) | -0.35725 | 0.623848 | -0.714931 | -0.280098 | 1.205719 | 0.574090 |
| Time (09:00) | 0.851712 | 1.278923 | -0.555546 | 0.156723 | 1.661110 | 1.119157 |
| Time (10:00) | 1.434443 | 1.638899 | -0.266045 | 0.860988 | 2.109240 | 1.524528 |
| Time (11:00) | 1.604866 | 1.429628 | 0.011957 | 1.447743 | 2.100046 | 1.664145 |
| Time (12:00) | 2.200384 | 1.104131 | 0.114004 | 1.711116 | 2.150003 | 1.821707 |
| Time (13:00) | 2.517669 | 0.711100 | 0.334918 | 2.321634 | 2.274665 | 1.668593 |
| Time (14:00) | 2.553804 | 0.628973 | 0.470670 | 2.982250 | 1.875389 | 1.317403 |
| Time (15:00) | 3.111274 | 0.697858 | 0.604793 | 2.611629 | 1.723889 | 1.046330 |
| Time (16:00) | 3.196034 | 0.609259 | 0.657485 | 2.029887 | 1.588638 | 0.860489 |
| Time (17:00) | 2.755565 | 0.516792 | 0.430146 | 1.628588 | 1.583145 | 0.519865 |
| Time (18:00) | 2.022618 | 0.301354 | 0.117382 | 0.902085 | 1.515682 | 0.436791 |
| Time (19:00) | 0.938271 | -0.035513 | -0.012613 | 0.500101 | 1.504069 | 0.603502 |
| Time (20:00) | -0.07934 | -0.095565 | 0.094742 | 0.230705 | 0.720162 | 0.199800 |
| Time (21:00) | -0.46764 | -0.173264 | -0.040348 | 0.079239 | 0.144805 | -0.101964 |
| Time (22:00) | -0.41889 | -0.198443 | 0.033351 | -0.016014 | -0.136991 | -0.243449 |
| Time (23:00) | -0.28498 | -0.158937 | -0.024441 | -0.062353 | -0.131455 | -0.206334 |
| Wind× Month(May) | 1 | 1 | 1 | 1 | NA | NA |
| Wind× Month(June) | 0.140528 | 0.090745 | -0.042541 | 0.047163 | 1 | 1 |
| Wind× Month(July) | 0.088279 | -0.098361 | -0.022605 | -0.042760 | 0.067163 | -0.000569 |
| Wind× Month(Aug) | -0.00095 | 0.054973 | 0.067002 | -0.136178 | 0.111277 | -0.039945 |
| Wind× Month(Sept) | -0.06374 | 0.028738 | -0.008487 | -0.136344 | 0.266283 | -0.172370 |
| Wind × Month(Oct) | -0.22943 | -0.102619 | -0.016493 | -0.172637 | -0.073921 | -0.170430 |
| Solar radiation × Month(May) | 1 | 1 | 1 | 1 | NA | NA |
| Solar radiation × Month(June) | -0.00084 | -0.000575 | -0.001453 | -0.003432 | 1 | 1 |
| Solar radiation × Month(July) | 0.000068 | -0.001064 | -0.001990 | -0.004728 | 0.002018 | 0.001148 |
| Solar radiation × Month(Aug) | 6.66E-06 | -0.002641 | -0.001767 | -0.005463 | -0.001457 | -0.000571 |
| Solar radiation × Month(Sept) | -0.00036 | -0.003305 | -0.001643 | -0.007005 | 0.006644 | 0.005799 |
| Solar radiation × Month(Oct) | 0.000007 | -0.004518 | -0.000146 | -0.007133 | 0.000755 | 0.005474 |
| Rain × Humidity | 0.030168 | 0.016478 | 0.007892 | 0.012372 | 0.013222 | 0.013732 |
| Wind speed × solar radiation | -0.00045 | -0.000368 | 0.000337 | 0.000212 | -0.000554 | -0.000598 |
| Wind speed × height(lower) | 1 | 1 | 1 | 1 | 1 | 1 |
| Wind speed × height (mid) | 0.12571 | 0.029565 | -0.011841 | NA | -0.023922 | -0.128821 |
| Wind speed × height (upper) | -0.17925 | 0.029798 | -0.046153 | 0.021786 | -0.013419 | -0.186326 |
| **Adjusted R^2^** | **0.9043** | **0.903** | **0.961** | **0.904** | **0.871** | **0.891** |

# **Figure S2:** The relationship between predicted temperature, solar radiation, wind speed, humidity, precipitation, DMI temperature, hour of the day and month at different microclimatic habitats at 13:00 in June. In the first six graphs, the DMI temperature was fixed at 15⁰C and all values were fixed at their respective median, in the last graph DMI temperature varied (from 0 to 35) and all values were fixed at their respective median.

**Supplementary document S3: (Model validation)**

*Method:*

Compare observed and predicted temperatures:

To validate the model, we divided the dataset into two equal halves: the first half contained the data of odd week numbers of the study period (week 19 to 43) and the second half contained the even week numbers of the study period (week 18 to 44). We then created a multiple linear regression model using the first dataset (odd week), and used this model to predict the hourly temperatures for the second dataset (even weeks). Finally, we plotted the observed and predicted temperatures of even weeks (Appendix Figure S3.1) and the residuals of the microclimatic models (Appendix S3.2). The dry meadow at Strødam and the horse and cattle fields at Faxe were similar habitats. To compare how our models behaved independently of location, we used the model developed for the dry meadow in Strødam to predict the microclimatic temperature of the cattle and horse fields at Faxe, and compared the observed and predicted temperatures.

*Results:*

Compare observed and predicted temperatures:

The predicted temperatures could explain 95 - 97% of the variation in the observed temperature in all microclimatic habitats (R^2^ = 0.95 - 0.97), and had a correlation coefficient of 0.92 - 0.98 with the observed microclimatic temperature. The residuals from the models were randomly distributed around zero throughout the period (Appendix S3.2). The root mean square error (RMSE) we observed in our microclimatic model was 2.17 for dry meadow, 1.87 for hedges, 0.87 for forest/trees, 1.44 for wet meadow, 2.10 for the cattle field, and 2.20 for the horse field. The predicted temperature generated by the dry meadow model could explain 85% of the variation in the observed temperatures in the cattle field (R^2^ = 0.85) and 83% of the variation in the observed temperatures in the horse field (R^2^ = 0.83; Appendix S3.3).

# **Figure S3.1:** The observed versus predicted microclimatic temperature during even weeks of six habitats (upper height), based on a microclimatic temperature model of odd weeks only, May – October 2015. Data from cattle and horse fields were available from 4^th^ June - 31^st^ October 2015, whereas data from other habitats were available from 1^st^ May – 31^st^ October 2015. The predicted temperature closely matched the observed temperature (correlation coefficient = 0.92 - 0.97).

**Figure S3.2:** The residuals of observed and predicted hourly temperatures of our microclimatic model for dry meadow, hedges, forest/trees, wet meadow and cattle and horse fields, May - October 2015, Denmark. The residuals are randomly distributed around zero.

|  |
| --- |

**Figure S3.3:** Comparison of the observed temperature (Obs.) and predicted microclimatic temperature (Pred.) at cattle and horse fields based on the model of dry meadow at Strødam (from the upper logger), June – October 2015. The predicted temperature at cattle and horse field of Faxe could explain 83% and 85% variation, respectively in the observed temperature in those habitats based on the model of the dry meadow at Strødam, Denmark.

**Figure S4:** The relationship between temperature, the development rate of pathogens and the blood meal digestion rate in mosquitoes and *Culicoides*. The threshold of pathogen development was between 10⁰C (Schmallenberg) and 19⁰C (dengue).


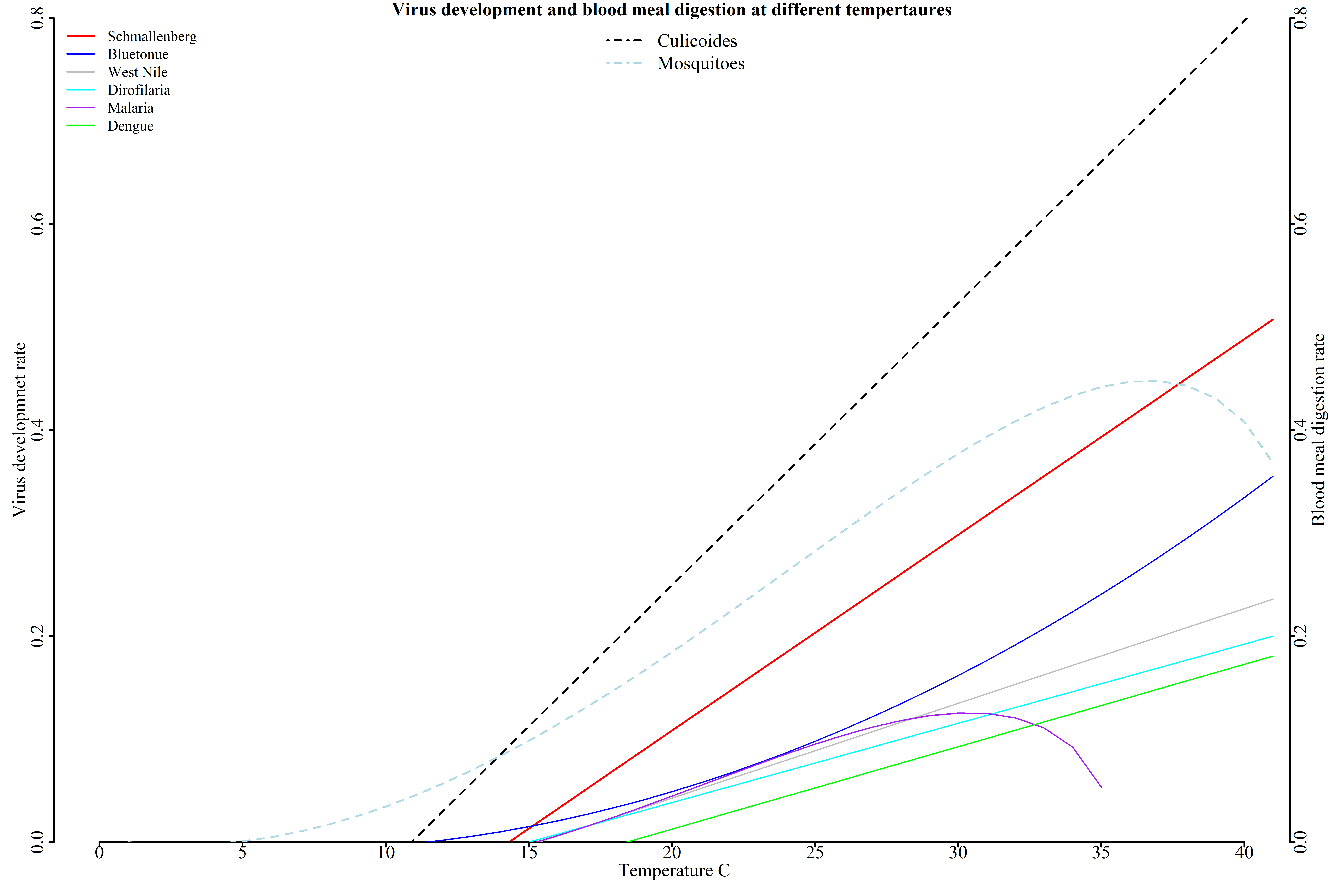


**Figure S5:** The weighting of each day of the month used in the linear regression model for converting DMI temperatures to microclimatic temperatures. The first 15 days of May and the last 15 days of October had no effects from neighboring months (as no data were available from April and November in our model) and therefore had a weight of 1. Each day of the study period had a cumulative weight of 1.
